# Association between Serum Iron and the Severity of Coronary Artery Disease

**Published:** 2013-09-01

**Authors:** Babak Bagheri, Mohammad Shokrzadeh, Vahid Mokhberi, Soheil Azizi, Alireza Khalilian, Negin Akbari, Valiallah Habibi, Keyvan Yousefnejad, Sasan Tabiban, Maryam Nabati

**Affiliations:** 1 Department of Cardiology, Faculty of Medicine, Mazandaran University of Medical Sciences, Sari, IR Iran; 2 Department of Toxicology, Faculty of Pharmacy, Mazandaran University of Medical Sciences, Sari, IR Iran; 3 Department of Medical Technology, Faculty of Medicine, Mazandaran University of Medical Sciences, Sari, IR Iran; 4 Department of Statistics, Faculty of Medicine, Mazandaran University of Medical Sciences, Sari, IR Iran; 5 Department of Cardiology, Mazandaran University of Medical Sciences, Sari, IR Iran; 6 Student Research Committee, Mazandaran University of Medical Sciences, Sari, IR Iran

**Keywords:** Coronary Artery Diseas, Iron, Risk Factors

## Abstract

**Background:**

Coronary Artery Disease (CAD) is the most important cause of mortality in the world. About half of cardiovascular risk factors have not been completely understood. Oxidation of LDL by oxidants such as iron plays a central role in atherogenesis. As a result, evaluation of the iron stores is important in the risk evaluation of the atherosclerotic disease.

**Materials and Methods:**

This cross sectional study was performed on 337 patients with chronic stable angina hospitalized in Sari heart center, Mazandaran University of Medical Sciences from February 2010 to July 2012. Coronary angiography was performed and the angiograms were evaluated by two cardiologists. Moreover, blood samples were collected after a 14-hour fast immediately before the coronary angiography in order to measure the total cholesterol, HDL- cholesterol, and glucose. The patients were divided into four groups to evaluate the severity of Coronary Artery Disease (CAD) according to Syntax scoring system.

**Results:**

The study results revealed a significant difference among the four study groups regarding the iron serum level. It was significantly higher in the sever atherosclerosis group compared to the normal (P=0.0122), mild (P=0.023), and moderate CAD groups (P<0.001).

**Conclusions:**

The findings indicated that the serum level of iron was higher in the atherosclerotic patients and increased with the severity of CAD. Therefore, a basic relationship probably exists between the serum iron level and CAD. Further prospective and experimental studies are needed to confirm the association between the iron status and atherosclerosis.

## 1. Background

Cardiovascular disease is epidemic at present and by 2020, it will be the single most important disease in the world in the terms of mortality, morbidity, disability, and economic loss. This chronic disease has also an enormous impact on the quality of life (1). Cardiovascular risk factors have not been completely understood yet. Coronary artery calcification is recognized as an independent predictor of cardiovascular disease and mortality (2, 3). The relationship between iron status and atherosclerosis has long been a topic of debate in the literature. Currently, there is no consensus in the medical literature regarding a causal relationship between the two. To date, the vast majority of studies have focused on iron burden with respect to a hypothesized role, whether direct or indirect, in the onset and/or progression of CAD (4). A putative role of iron in atherosclerosis has been proposed in part for the sex differences in cardiovascular disease (CVD) and for the higher incidence of heart disease in older individuals related to age-associated increase in iron stores (5). Via iron-dependent enzymatic processes, iron has a pivotal role in maintaining the cellular function and integrity and mediates a variety of metabolic processes. However, careful homeostasis is also critical as both iron-deficient (e.g. skeletal muscle dysfunction) and overload (e.g. hemochromatosis) states can be deleterious to the human body (6).

For the first time, Sullivan (7) suggested time that iron stores in human body may be positively correlated with the risk of coronary heart disease. According to his hypothesis, production of free radicals that are subsequently modified to low density lipoprotein cholesterol (LDL) is important in the development of atherosclerosis and iron may stimulate the catalyzing of oxidation reactions that produce free radicals (8, 9).

Although the vast majority of basic and animal researches have been largely supportive of the role of iron status in atherosclerosis, human studies have been equivocal (4). Moreover, several studies have investigated the role of iron in the development of CVD, but have come to inconsistent results.

Thus, the present study aims to investigate the relationship between serum levels of iron and the severity of CAD measured by Syntax score.

## 2. Patients and Methods

The present cross sectional study was performed on the patients hospitalized in Sari heart center, Mazandaran University of Medical Sciences from February 2010 to July 2012. Written informed consent was obtained from all the participants according to the criteria of the Ethical Committee of Mazandaran University of Medical Sciences.

The sample consisted of 337 patients with chronic stable angina each of whom had been admitted for diagnostic coronary angiography for typical indications, such as evaluation of stable exertional angina.

The patients who had a history of infectious diseases in the recent two months, collagen vascular disease, and recent cardiac events were excluded from the study.

Coronary angiography was performed by the Judkins technique through femoral artery access and the angiograms were evaluated by two cardiologists who were blinded to the study plan.

The study patients were divided into four groups to evaluate the severity of Coronary Artery Disease (CAD) according to Syntax scoring system.

The diagnosis of CAD was based on the presence of 50% luminar diameter stenosis of the major epicardial coronary artery which was determined in a standard manner during the coronary angiograms (1.5 mm less severe lesions should not be included in the SYNTAX score). The percent diameter stenosis was not considered in the algorithm and distinction has been made only between occlusive (100% diameter stenosis) and non occlusive (50-99% diameter stenosis) disease. Besides, multiplication factors of 2 and 5 were used for non-occlusive and occlusive lesions, respectively. Furthermore, CAD was categorized into three groups of mild for 0-22 scores, moderate for 23-32 scores, and severe for 33 and higher scores (10, 11).

### 2.1. Cardiac Risk Factors

Cardiovascular risk factors, including age, sex, systolic and diastolic blood pressure, smoking status, history of dyslipidaemia, and diabetes, were assessed for each subject.

Dyslipidaemia was defined as the Total Cholesterol (TC) to High Density Lipoprotein (HDL) more than 4. In addition, Hypertension (HT) was defined as a systolic blood pressure above 140mmHg, diastolic blood pressure above 90mmHg, or current use of antihypertensive medication. Besides, Diabetes Mellitus (DM) was defined as a known history of DM, fasting blood glucose of 126 mg/dl, GTT greater than 200 mg/dl, or treatment with insulin or oral hypoglycemic agents. Moreover, different categories of cigarette smoking status were defined according to World Health Organization (WHO) guidelines (12). Daily smoker was defined as an individual who smoked cigarettes at least once a day. Occasional smoker was the one who smoked cigarettes but not every day. On the other hand, ex-smoker was a former daily or occasional smoker who currently did not smoke. Finally, never smoked was defined as a person who never smoked before or smoked too little in the past.

### 2.2. Biochemical Evaluation

Blood samples were collected after a 14-hour fast immediately before the coronary angiography was started for measurement of total cholesterol, HDL- cholesterol, and glucose. The samples were centrifuged at 3000g for 10 min at ambient temperature. The obtained serum was separated and frozen at -80 C until the time of analysis. Iron was measured by Microparticle Enzyme Immunoassay (MEIA) technology (Abbott AxSym, Abbott Park, IL, USA).

### 2.3. Statistical Analyses

The study data were analyzed using the SPSS-16 software. Baseline demographic and laboratory data were presented as mean ± SD for continuous variables and as frequencies for discrete variables. Parametric and non parametric data were compared between the study groups using one-way ANOVA and chi-square tests. Besides, the mean difference of iron level was compared among the four groups using one-way ANOVA. P<0.05 was considered as statistically significant.

## 3. Results

Table 1 summarizes the demographic data of the study subjects.

No significant difference was found among the four groups regarding the smoking habits (P=0.682), occurrence of DM (P=0. 843) and HT (P= 0. 552), serum TC/ HDL, and blood glucose levels.

The main results of the study are summarized in Table 1. In this study, the serum level of iron was significantly increased with the severity of atherosclerosis. It was significantly higher in the severe atherosclerosis group (33 and higher Syntax scores) compared to the mild and moderate CAD groups (P<0.001).

**Table 1.** Demographic, Para-Clinical, and Clinical Data of the Subjects

Characteristic	Normal Coronary (n=87) (mean±SD)	Mild CAD (n=82) (mean±SD)	Moderate CAD (n=80) (mean±SD)	Severe CAD (n=88) (mean±SD)	*P *value
**FBS**	129.42±60.09	131.35±60.28	141.57±79.52	134.29±59.70	0.184
**Systolic BP(mmHg)**	118.46±8.49	120.27±8.13	120.77±9.17	118.78±7.48	0.345
**Diastolic BP (mmHg)**	75.77±5.36	74.66±5.005	75.61±5.71	74.39±5.20	0.817
**TC/HDL**	4.61±1.27	4.56±1.17	4.56±1.84	5.14±1.49	0.734
**Age(year) **	7.74±53.46	9.30±59.83	8.99±58.6	58.75±9.53	0.021
**Daily cigarette smokers (n%)**	7 (8.7)	4 (4.5)	3 (3.7)	3 (3.4)	0.561
**Plasma iron (mg/l)**	1.9±9.4	2.5 ±13.2	2.1 ±27.2	3.3 ±28.5	0.001
**Sex:female (n (%))**	33 (63.5%)	75 (51.4%)	45 (45.9%)	14 (34.1%)	0.032

Moreover, the serum level of iron in the normal coronary group was 9.4±1.9 mg/L. Sheffe test results showed that the iron level was significantly higher in the severe atherosclerosis group in comparison to the normal (P= 0.0122), mild (P=0.023), and moderate CAD groups (P<0.001).

## 4. Discussion

CAD is a leading cause of mortality, morbidity, and disability in Iranian population. It accounts for nearly 50% of all the annual CAD-related deaths in IR Iran (13). CAD has been associated with several risk factors, including sex, age, elevated blood cholesterol, diabetes mellitus, cigarette smoking, hypertension, and atherosclerosis (14). Several trace elements have also been implicated in the pathogenesis of CAD (15). Iron plays an important role in production of free radicals and peroxidation of lipids and myocardial ischemic damage (16). The current cross sectional study showed a significant difference among the study groups regarding the iron level and serum iron level was lower in the normal coronary group compared to the total atherosclerotic groups. In addition, the serum level of iron was increased with the severity of atherosclerosis and the highest serum iron level was observed among the severe atherosclerosis patients (33 and above Syntax scores).

The relationship between iron status and atherosclerosis has long been a topic of debate in the literature.

Steinberg showed that reduced iron played a role in the peroxidation of lipids (17). In a study, Ascheivo et al. found a relationship between iron consumption and infarction risk among the men whose diet did not include vitamin E (18). Furthermore, Salonen et al. showed a two-fold increased risk of myocardial infarction in the subjects with above 200 g/l ferritin levels (19). In the same line, Tuomainen revealed that the mean TfR/ferritin ratio was significantly higher in males who experienced myocardial infarction compared to the controls subjects (20). In a nested case-control analysis, Klipstein Grobusch et al. also confirmed a positive correlation between serum ferritin and risk of myocardial infarction, which was more pronounced in conjunction with increased cholesterol levels and smoking status (21). Moreover, Lauffer in a review of data from 11 countries found that the ferritin serum cholesterol product demonstrated the best correlation with CAD mortality rates in both sexes (22).

However, some studies have suggested that iron does not play a major role in development of CAD. More recent data from the National Health and Nutrition Examination Study (NHANES II) did not demonstrate a relationship between ferritin and cardiovascular mortality (23). On the other hand, Knulman et al. in a cohort study reported that sufficient evidence was not found regarding ferritin level being a risk factor for cardiovascular disease (24). Magnusson and Pilote et al. did not find any association between serum ferritin and the risk of myocardial infarction (25, 26). The results of these epidemiological studies are inconsistent and do not definitively show whether high iron level is a cardiac risk factor.

## 5. Conclusion

In conclusion, the findings of the present study showed elevated serum iron concentrations to be associated with increased syntax score and athrosclerosis severity. The present data suggested that iron may play a role in the incident of atherosclerosis. In this study, iron level was measured for once and error of measurement should be considered. Thus, further studies are required to be conducted on the issue in a clinical model of early and advanced stages of atherosclerosis.
